# Indirect Calorimetry Performance Using a Handheld Device Compared to the Metabolic Cart in Outpatients with Cirrhosis

**DOI:** 10.3390/nu11051030

**Published:** 2019-05-08

**Authors:** Lauren Schock, Louisa Lam, Puneeta Tandon, Lorian Taylor, Maitreyi Raman

**Affiliations:** 1 Cumming School of Medicine, University of Calgary, 3330 Hospital Drive NW, Calgary AB T2N 4N1, Canada; lauren.schock@ucalgary.ca; 2 Sheldon M. Chumir Health Centre, Alberta Health Services, 4th floor, cubicle 4256, 1213 4th Street SW, Calgary AB T2R 0X7, Canada; louisa.lam@ahs.ca; 3 Division of Gastroenterology (Liver Unit), Zeidler Ledcor Center, 130 University Campus, Edmonton AB T6G 2X8, Canada; ptandon@ualberta.ca; 4 Division of Gastroenterology, University of Calgary, 6D33 TRW Building, 3280 Hospital Drive NW, Calgary AB T2N 4N1, Canada; lorian.taylor@ucalgary.ca

**Keywords:** malnutrition, cirrhosis, indirect calorimetry, MedGem^®^, VMax, nutrition

## Abstract

Addressing malnutrition is important to improve health outcomes in outpatients with cirrhosis, yet assessing energy requirements in this population is challenging. Predictive equations of resting energy expenditure (REE) are thought to be unreliable, and traditional indirect calorimetry is expensive and infrequently available for clinical use. The accuracy of REE predictions using a MedGem^®^ handheld indirect calorimeter, the Harris Benedict Equation (HBE), the Mifflin St. Jeor equation (MSJ), and the gold standard Vmax Encore^®^ (Vmax) metabolic cart was compared. The REE of cirrhotic pre-liver transplant outpatients was analyzed using each of the four methods. Agreement between methods was calculated using Bland–Altman analysis. Fourteen patients with cirrhosis participated, and were primarily male (71%) and malnourished (subjective global assessment (SGA) B or C 64%). Lin’s concordance coefficient (ρC) for MedGem^®^ vs. Vmax demonstrated poor levels of precision and accuracy (ρC = 0.80, 95% confidence interval 0.55–0.92) between measures, as did the HBE compared to Vmax (ρC = 0.56, 95% confidence interval 0.19–0.79). Mean REE by MedGem^®^ was similar to that measured by Vmax (−1.5%); however, only 21% of REE measures by MedGem^®^ were within ±5% of Vmax measures. Wide variability limits the use of MedGem^®^ at an individual level; a more accurate and feasible method for determination of REE in patients with cirrhosis and malnutrition is needed.

## 1. Introduction

Malnutrition is prevalent among patients with cirrhosis for multiple reasons including inadequate energy intake, impaired gut motility, nutrient malabsorption, increased protein losses, and a metabolic shift to gluconeogenesis and fatty acid oxidation resulting in an accelerated state of starvation [1,2,3]. The loss of skeletal muscle and fat mass is an independent risk factor for increased morbidity and mortality [4,5]. Clinical consequences of malnutrition can include infection, compromised immune function, progression of liver dysfunction, hepatic encephalopathy, and increased mortality both before and after liver transplantation [6,7,8,9]. Optimizing nutrition, using a multi-disciplinary nutrition intervention, can improve patient outcomes and quality of life [10,11,12].

Assessment of nutritional status and identifying adequate energy requirements are complicated in cirrhosis. Predictive equations are often used to estimate the resting metabolic rate (RMR), the energy expended to sustain homeostasis at rest [13]. Resting energy expenditure (REE) refers to RMR measured under standardized testing conditions. REE makes up the majority of total energy expenditure (TEE) for a patient, which is comprised of REE as well as additional calories to account for physical activity and activities of daily living [13]. Predictive equations rely on accurate weight measurements, which in cirrhosis, may be obscured by edema or ascites [10,14]. Predictive equations do not account for differences in body composition, underlying clinical conditions, and metabolic disturbances such as the shifts in substrate metabolism associated with cirrhosis [15]. The Harris Benedict equation (HBE) and Mifflin St. Jeor (MSJ) equation are commonly used in clinical practice, and they have been found to systematically underestimate RMR in patients with cirrhosis compared to the metabolic cart, the measurement tool with the highest levels of validity and reliability [15].

Consensus guidelines recommend using a nutrition prescription that provides 28–37.5 kcal/kg/day for patients with cirrhosis [16]. This weight-based recommendation was based on consensus; as quality of evidence is limited, it is reasonable to surmise that physical activity, disease severity, and need for weight gain were factored into this recommendation. A major concern with using this weight-based recommendation without individualization is the risk of overfeeding, both in cirrhotic patients with obesity or ascites and in patients of normal body mass index (BMI) with preserved lean body mass.

The measurement tool with the highest levels of validity and reliability for the assessment of REE is a metabolic cart, such as the Vmax Encore^®^ (Vmax) [15,17,18,19,20,21,22,23]. Vmax has been validated using varying methodology, most commonly through comparison to the Deltatrac metabolic cart (no longer in production); the variation between devices has been reported as 4–8%, and reliability for inter-device measurement with Vmax as 3–5% [19,21]. Metabolic carts use calibrated standardized gases to measure both oxygen consumption (VO_2_) and carbon dioxide output (VCO_2_) to determine the respiratory quotient and subsequently mathematically derive the REE for a given patient [13]. Metabolic carts have some limitations to measuring REE in outpatient settings as they are large, expensive, and require special training to administer and interpret [24]. A novel method to measure REE is the handheld indirect calorimeter (brand name MedGem^®^ (Microlife Home Medical Solutions Inc., Golden, CO, USA)). The handheld indirect calorimeter has fewer limitations than the larger metabolic cart; it is easier to administer, portable, and much less expensive. Notably, MedGem^®^ does not use a gas exchange system to determine respiritory quotient (RQ) for the individual, instead only measuring oxygen consumption and using a fixed respiratory quotient to calculate REE. MedGem^®^ has been found to be an accurate and reliable method to predict REE in healthy and overweight (BMI M = 26.5) patient populations [25]. In four comparative studies, MedGem reliability showed an intraclass correlation coefficient (ICC) = 0.97–0.98, and correlation to Douglas Bag method was shown as ICC = 0.91–0.97) [26]. In two studies among patients with malnutrition secondary to cancer and anorexia nervosa, the MedGem^®^ was found to be biased towards a lower REE measurement compared to two different metabolic carts (REE variation M = 9–10%) [27,28]. These studies concluded further valuation in a malnourished population was necessary before MedGem^®^ could be recommended in clinical practice for use in individual patients [27,28]. In the only study to investigate the use of MedGem^®^ among 25 patients with cirrhosis, Glass et al. (2012) identified that HBE consistently underpredicted REE by an absolute difference of 18.6% (±2.5) in 16 patients and overpredicted by an absolute difference of 6.8% (±1.8) in nine patients. Mifflin St. Jeor underpredicted REE by 21.1% (±3.4) in 14 patients and overpredicted REE by 9.3% (±21.0) in 11 patients. The handheld indirect calorimeter underpredicted REE by 11.0 to 19.1% (±3.3, 3.5) in 11 patients and overpredicted REE by 13.5% to 17.6% (±2.7, 4.3) in 15 and 14 patients, respectively, dependent on the testing environment [15].

The primary objective of this study was to compare the accuracy of REE measurement using a MedGem^®^ handheld indirect calorimeter compared to a metabolic cart in patients with cirrhosis. Accuracy was defined as the percentage of REE estimates that fell within a five percent range above or below REE values measured via the Vmax metabolic cart. The secondary objective was to evaluate the accuracy of the Harris Benedict equation (HBE) and Mifflin St. Jeor (MSJ) equation as compared to the REE calculated by the metabolic cart. 

## 2. Materials and Methods

Research ethics approval to complete this study was obtained through the University of Calgary Conjoint Health Research Ethics Board. Participants were provided with verbal and written information about the study before providing their written consent.

### 2.1. Patients

The sample population was composed of 15 patients with cirrhosis from the Malnutrition Clinic, a specialty outpatient clinic for pre-liver transplant nutrition assessment and management in Calgary, Alberta. Patients were eligible to participate if they had a current diagnosis of liver cirrhosis and were awaiting liver transplantation assessment. Demographic data collected included age, sex, height, estimated weight, estimated dry BMI, aetiology of cirrhosis, model for end-stage liver disease (MELD) score, presence of ascites and malnutrition status determined using subjective global assessment (SGA) (Table 1). SGA is a validated tool used to classify patients as mildly, moderately, or severely malnourished [29].

Patients were excluded if they had received a general anesthetic within the previous 24 h, were discharged from hospital within 24 h, were within six hours of hemodialysis, used supplemental oxygen therapy, had claustrophobia, nausea, vomiting, were unable to hold the MedGem^®^ calorimeter, or were unable to tolerate lying supine for the required time. Patients were required to meet the procedure criteria that included a fasted state for eight hours, avoidance of exercise or caffeine within four hours, and avoidance of nicotine, allergy or cold medications, or herbal supplements within one hour of testing.

### 2.2. Procedure

REE was measured using the Vmax Encore^®^ metabolic cart (Vyaire Medical Inc., Yorba Linda, CA, USA), which was calibrated according to manufacturer’s instructions prior to each use. A clear plastic canopy is placed over the participant’s head, attached to the Vmax by disposable plastic tubing. The Vmax Encore^®^ measures VCO_2_, and VO_2_ (litres per minute) and thus calculates the respiratory quotient (RQ) and REE. Vmax testing was carried out for up to 30 min. The first five minutes of testing were discarded in all cases. After the initial, discarded five-minute period, once five minutes of steady state were achieved patient testing was concluded; if achieved, all five minutes of steady state data were included in the analysis. Steady state is defined as a time period in which the mean VO_2_ and VCO_2_ are changed by less than 10% [30]. For those who did not achieve five minutes of steady state data collection, testing was continued to 30 min; the first five minutes of data were discarded, and the latter 25 minutes of data were averaged for analysis. Data analysis was carried out through a trained user and the Vmax machine. This protocol was in accordance with best practice guidelines for Vmax use [18,21,22,30,31]. The MedGem^®^ hand-held indirect calorimeter is a self-calibrating device. It measures VO_2_ (mL per minute) only and uses a fixed RQ of 0.85 to calculate REE. Patients wore a nose clip and disposable mouthpiece connected to the HHRC, which they supported with one arm for the measurement period. Measurement took approximately five to ten minutes, with the end time automatically determined by the device.

Eligible patients were assessed by the Vmax Encore^®^ and the MedGem^®^ indirect calorimeters consecutively. Completion of Vmax and MedGem^®^ tests were conducted in randomly assigned alternating order to minimize bias. Exceptions to randomly assigned testing order were made in three instances where the Vmax failed to calibrate within a one-hour time frame leading to MedGem^®^ testing first. In each of these instances the Vmax was calibrated, and testing completed before the end of the appointment.

Patients rested for 20 min prior to the first test, and remained in the recumbent, relaxed position between testing [18,21,22]. The second test was carried out within a 5–15 min period after the first test was completed [17,22]. Appointments were conducted in the morning between 08:00 and 12:00 h; morning hours were chosen considering fasting requirements.

All participants were studied in the supine position, recumbent with torso inclined at approximately 30–45 degree angle, in accordance with best practice guidelines [15]. Slight variations in position were made to assure the comfort of each participant, and to minimize movement during the testing period [21]. When using the MedGem^®^ device, the arm holding the device was propped in order to minimize the effect of arm activity; this position has been found to be insignificant on final REE [15]. No other changes in positioning were made when switching in between Vmax and MedGem^®^.

HBE and MSJ were calculated for each patient based on their weight on the day of the appointment, using their estimated dry weight as evaluated by the trained researcher. HBE values for the purpose of this study do not include a disease cofactor or activity cofactor as they are used to calculate REE versus TEE.

### 2.3. Statistical Analysis

After collection, data from one participant were excluded before analysis due to extreme deviation in measured Vmax REE compared to both REE determined from the other three methods, as well as significant deviation from clinically expected values. We are not aware of any distinguishing characteristics of this patient that might have accounted for this error.

Data are reported as mean ± standard deviation (SD). Analysis of variation with Student’s two-sided paired *t*-test was used to compare mean differences between testing methods, with the statistical significance level set at α = 0.05.

Lin’s concordance correlation coefficient (ρC) was used to examine the relationship and level of agreement of Vmax to MedGem^®^, Vmax to HBE, and Vmax to MSJ [32]. A scale for interpreting relationships between datasets using ρC values suggests an almost perfect relationship is ρC >0.99, a moderate relationship is ρC 0.95–0.99, a fair relationship is ρC 0.90–0.95, and a poor relationship is < 0.90 [32].

Bland–Altman plots depict the difference between paired measurements to the mean of the pair, and bars to represent limits of agreement depict the agreement between methods for determining REE [33]. The limits of agreement were set ± 1.96 standard deviations from the mean with another line added representing a 5% kcal over- or underestimation, based on literature standard for critical value difference in REE measuring devices [15,26,27].

## 3. Results

The mean age of the 14 patients was 53.5 ± 9 years, with a total of 10 (71%) men. Two-thirds of the study patients were malnourished (SGA B or C). Patient demographics, aetiology of cirrhosis, disease characteristics, and nutrition status are reported in Table 1. The mean measured REE using the Vmax was 1472 kcal/day (SD ± 279). Mean REE assessment by MedGem^®^ underpredicted REE by 1.3% compared to Vmax. HBE underpredicted REE by 4.2%, and MSJ underpredicted REE by 6.6%. The mean REEs are reported in Table 2.

**Figure 1:**
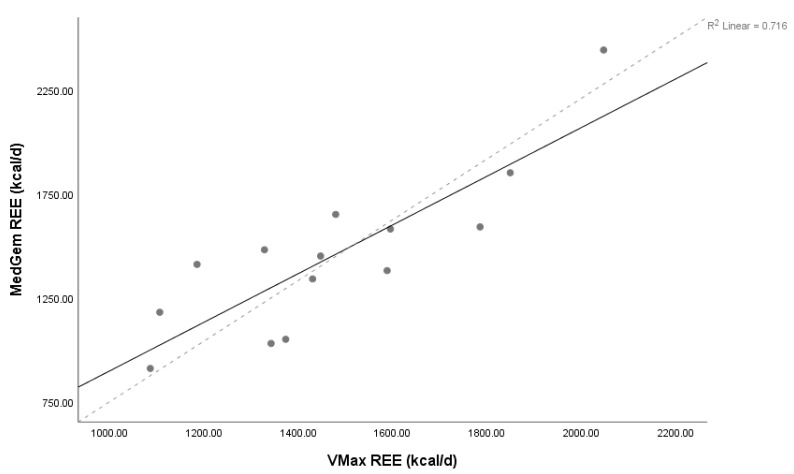
Concordance between MedGem and Vmax. Lin’s concordance correlation coefficient (ρC) = 0.80, 95% confidence interval 0.55–0.92, *n* = 14. Solid line represents line of identity, dashed line represents line of best fit. Abbreviations: REE = resting energy expenditure, d = day.

**Figure 2:**
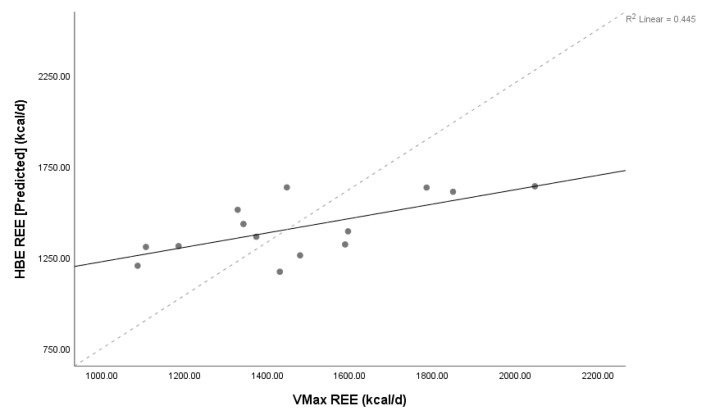
Concordance between the Harris Benedict equation and Vmax. Lin’s concordance correlation coefficient (ρC) = 0.56, 95% confidence interval 0.19–0.79, *n* = 14. Solid line represents line of identity, dashed line represents line of best fit. Abbreviations: REE = resting energy expenditure; HBE = Harris Benedict equation, d = day.

**Figure 3:**
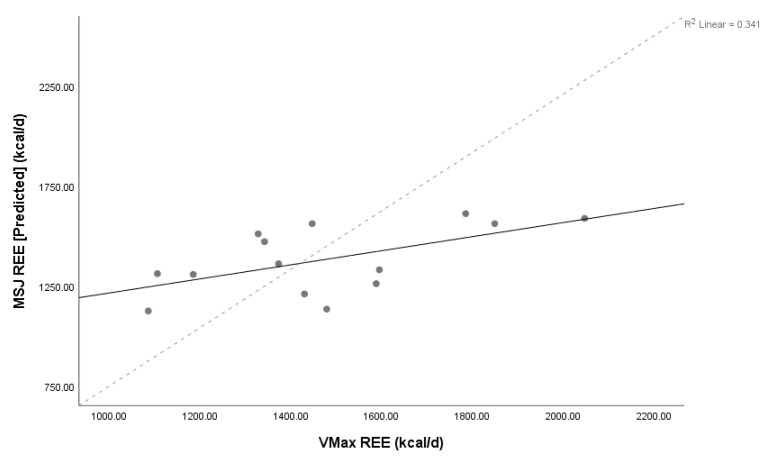
Concordance between Mifflin St Jeor equation and Vmax. Lin’s concordance correlation coefficient (ρC) = 0.47, 95% confidence interval 0.07–0.75, *n* = 14. Solid line represents line of identity, dashed line represents line of best fit. Abbreviations: REE = resting energy expenditure; MSJ = Mifflin St. Jeor equation, d = day.

Lin’s concordance coefficient (ρC) for MedGem^®^ vs. Vmax demonstrated poor levels of precision and accuracy (ρC = 0.80, 95% confidence interval 0.55–0.92) between measures, as did HBE and Vmax (ρC = 0.56, 95% confidence interval 0.19–0.79), and MSJ and Vmax (ρC = 0.47, 95% confidence interval 0.07–0.75). The relationship between MedGem^®^ and both HBE and MSJ were also poor (ρC = 0.43, 95% confidence interval 0.09–0.67; ρC = 0.35, 95% confidence interval –0.01–0.63, respectively). Scatter plots of MedGem^®^ vs. Vmax, HBE vs. Vmax, and MSJ vs. Vmax REE provide a visual depiction of the correlation of each data point (Figure 1, Figure 2 and Figure 3). Analyses using Shapiro–Wilk (W) demonstrated non-significant results for normality for REE reported by Vmax, MedGem^®^, HBE, and MSJ (W = 0.96, *p* = 0.68; W = 0.92, *p* = 0.22; W = 0.91, *p* = 0.15; W = 0.92, *p* = 0.25, respectively) demonstrating data were normally distributed and appropriate for Bland–Altman analyses.

Twenty-one percent (*n* = 3) of MedGem^®^ measurements of REE fell into the acceptable ±5% kcal limits as compared to Vmax REE; 43% (*n* = 6) of MedGem^®^ measurements of REE were underestimated by more than five percent, and 36% (*n* = 5) of MedGem^®^ measurements of REE were overestimated by greater than five percent. Seven percent (*n* = 1) of HBE, and 14% of MSJ predicted REE fell into the acceptable ±5% kcal limits compared to Vmax REE. HBE underestimated REE by more than 5% kcal in 50% (*n* = 7), and overestimated REE by >5% in 43% (*n* = 6). MSJ both under- and overestimated REE by more than 5% in 43% (*n* = 6).

**Figure 4:**
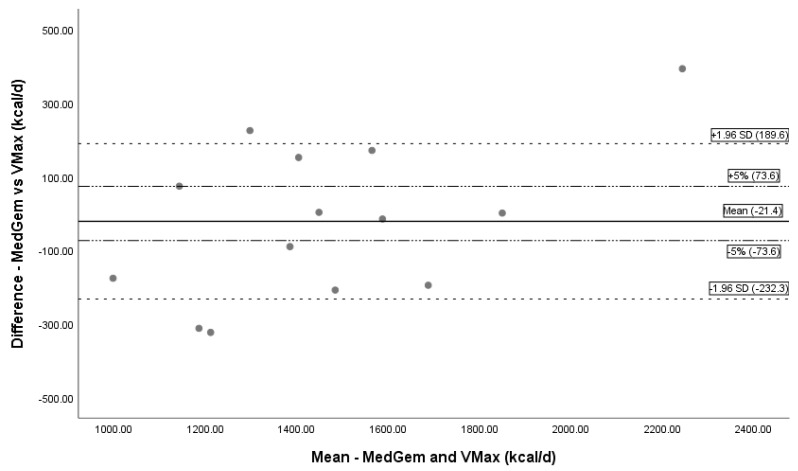
Bland–Altman plot Vmax and MedGem. Bland–Altman plot showing the mean bias and limits of agreement between MedGem and Vmax. The solid line represents mean bias (−21.4 kcal/day), a systematic undermeasurement of REE by MedGem. Dashed lines represent confidence intervals (± 1.96 SD). Dotted and dashed lines are a reference line for ±5%difference. Abbreviations: REE = resting energy expenditure; d = day.

**Figure 5:**
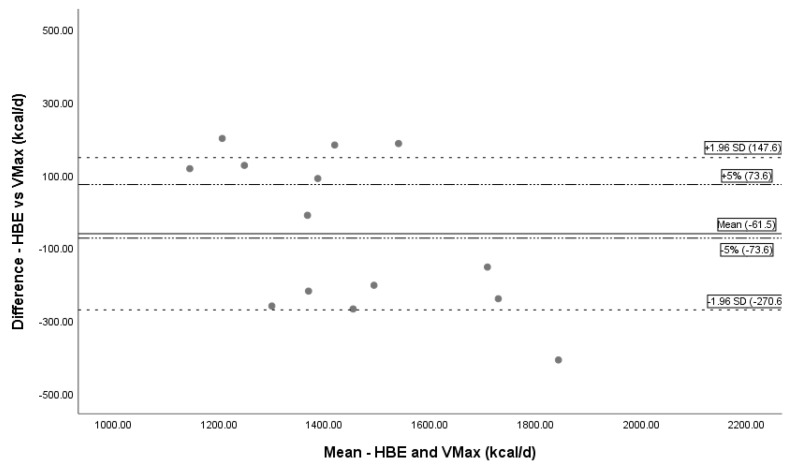
Bland–Altman Plot HBE and Vmax. Bland–Altman plot showing the mean bias and limits of agreement between Vmax and HBE. The solid line represents mean bias (−61.5 kcal/day), a systematic underprediction of REE by HBE. Dashed lines represent confidence intervals (± 1.96 SD). Dotted and dashed lines are a reference line for ±5% difference. Abbreviations: REE = resting energy expenditure; HBE = Harris Benedict equation, d = day.

**Figure 6:**
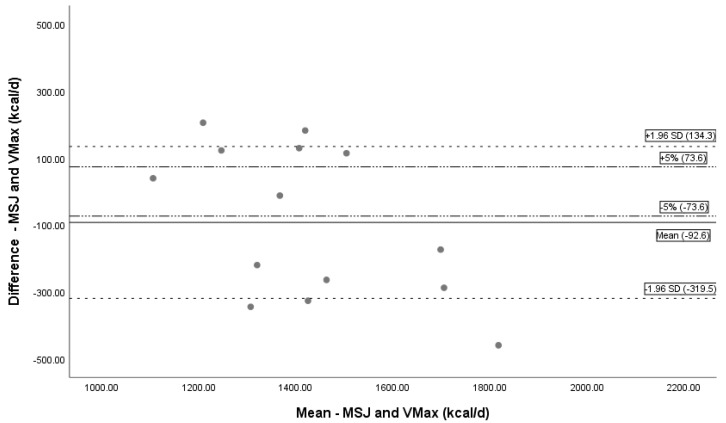
Bland–Altman Plot MSJ and Vmax. Bland–Altman plot showing the mean bias and limits of agreement between MSJ and Vmax. The black solid line represents mean bias (−92.6 kcal/day), a systematic underprediction of REE by HBE. Dashed lines represent confidence intervals (± 1.96 SD). Dotted and dashed lines are a reference line for ± 5% difference. Abbreviations: REE = resting energy expenditure; MSJ = Mifflin St. Jeor equation, d = day.

REE using MedGem^®^ underpredicted measured REE using Vmax by a mean kcal/day difference of 1.5% (−21.5 ± 211.0 kcal, *n* = 14) (Table 3). The mean difference was statistically significant (*p* < 0.01). Bland–Altman analysis depicts the systematic undermeasurement in REE and limits of agreement (Figure 4). REE calculated by HBE under predicted mean REE compared to Vmax by 4.2% (−61.5 ± 209.0 kcal, *n* = 14), and calculated by MSJ under predicted mean REE by 6.2% (−92.6 ± 226.9 kcal, *n* = 14). Both results were statistically significant (*p* = 0.01, *p* = 0.03 respectively); Bland–Altman analysis is depicted in Figure 5; Figure 6.

MedGem^®^ over- and underpredicted REE in similar numbers of patients. When MedGem^®^ underpredicted REE, values were 13% lower than Vmax (−188.4 ± 110.8 kcal, *n* = 7, *p* < 0.01). In contrast, MedGem^®^ overpredicted REE compared to Vmax by 10% (145.6 ± 138.2 kcal, *n* = 7, *p* = 0.02). HBE also over- and underpredicted REE in similar numbers of patients. HBE underpredicted REE by 13% (−220.3 ± 112.2 kcal, *n* = 8, *p* < 0.01), compared to overpredicting by 12% (150.2 ± 44.9 kcal, *n* = 6, *p* < 0.01). MSJ demonstrated a wider respective under and over prediction than other measures. MSJ underpredicted REE in fewer patients, by 20% (−261.1 ± 132.3 kcal, *n* = 5, *p* = 0.01), and overpredicted REE in the majority of patients, by 8% on average (132.1 ± 58.1 kcal, *n* = 9, *p* = 0.01) (Table 3).

When SGA categories were stratified, MedGem^®^ appeared to overestimate REE in SGA A patients by 5% and underestimate in SGA B patients (−6%) and SGA C patients (−5%). Only the SGA B category achieved significance (*p* = 0.04). After HBE values were stratified using SGA, there was a non-significant mean underestimation in all groups, with the highest degree of underestimation in the SGA A group (−7%), followed by SGA B (−2%) and SGA C (−3%). There was a similar, non-significant underestimation of REE by MSJ when stratified to SGA class; SGA A patients were underestimated by 10%, SGA B by 2%, and SGA C by 6%.

## 4. Discussion

The present study reports on the effectiveness of two measures of REE compared to the metabolic cart; the reference standard measure of REE. To our knowledge, this is the first study to describe the relationship between a handheld indirect calorimeter in outpatients with cirrhosis. Our findings demonstrate poor relationships between Vmax and MedGem^®^ (ρC = 0.80), Vmax and HBE (ρC = 0.56), and Vmax and MSJ (ρC = 0.47). Although MedGem^®^ demonstrated a superior relationship with Vmax than did HBE or MSJ, it was not found to be accurate on an individual patient level. Our chosen level of agreement was ±5% as is standard in similar literature. According to MedGem^®^, 21% of patients tested fell in this category, compared to only 14% of HBE MSJ and 7% of HBE REE values. Although MedGem^®^ demonstrated better agreement, all tools suggest a higher than acceptable level of disagreement for determining REE for the majority of patients in this study.

Mean REE by MedGem most closely approximated Vmax measurement (mean difference −21 kcal/day). MSJ underestimated REE by the largest margin, which was 93 kcal/day on average. Interestingly, MedGem^®^ indirect calorimetry underpredicted mean REE, but over- and under-predicted in an equal proportion of patients, therefore not supporting the presence of a systematic bias. When MedGem^®^ indirect calorimetry under-predicted REE, the magnitude of mean difference was nearly 190 kcal/day. This under-prediction is of concern due to magnitude, and largely observed in a malnourished subset of patients (SGA B and C). Similarly, using HBE and MSJ, the magnitude of under-prediction was of clinical relevance, approaching 220 kcal/day and 260 kcal/day respectively. Identifying patient or disease related factors that may predict REE under-prediction is of importance, as under-dosing the nutrition prescription may have deleterious consequences in this population at risk for or with active malnutrition. It is interesting to observe MedGem^®^ under-predicted mean REE in malnourished patients compared to well-nourished patients, although these results may have been due to chance. Our findings regarding MedGem^®^ were similar to those reported by Glass et al. (2012) in their validation study. Glass reported a mean difference of −28 kcal/day (95% CI = −150 to 94 kcal/day) between REE measured by MedGem^®^ and Vmax when tested in a quiet environment that was similar to our testing environment [15]. While the authors concluded the handheld calorimeter is a “valid, feasible” method, their study population may not have represented malnourished cirrhosis patients as they had a mean body mass index of 29.6 ± 7.7 with a range of 18.5–56.1 and 68% had ascites. The authors did not specify if reported BMI was based on current body weight or estimated dry weight. No other measures of nutritional status were reported [15]. Another major difference between studies was the outpatient population in our study, which may be associated with underlying differences in patient health status and activity levels compared to the hospitalized patients. In a similar validation study of MedGem^®^ in both healthy patients and patients with cancer, Reeves et al. (2005) also used 5% variation in REE as clinically acceptable disagreement in measurement as compared to REE measured by Vmax. The authors found that less than one-third of tested patients fell within the acceptable range, concluding that limits of agreement for MedGem^®^ versus traditional indirect calorimetry were too wide and provided poor clinical accuracy for the determination of REE at an individual level [27]. Considering that less than one-quarter of our patients fell within this limit, we find this level of disagreement to be higher than acceptable. A major difference between the handheld MedGem^®^ and traditional indirect calorimetry is the fixed RQ used in calculating the final REE with MedGem^®^, which may not accurately reflect metabolic shifts occurring in individuals with advanced liver disease. In comparison, Vmax measures both VCO_2_ and VO_2_ to calculate a RQ that is specific to each patient, thereby reflecting their current metabolic demands. A low RQ reflects a heavier reliance on fatty acid metabolism and gluconeogenesis, and has been documented in individuals with sarcopenia and cirrhosis, which has been interpreted as evidence of accelerated starvation [34,35,36,37]. Patients with severe malnutrition or advanced cirrhosis could be expected to have a progressively lower RQ, which could result in inaccuracies in REE calculated by MedGem^®^ [16]. This is generally supported by the trend in subgroup analysis whereby SGA A was overestimated by MedGem^®^ (+5%), whereas SGA B and C were underestimated (SGA B −6%; SGA C −5%); even though only the SGA B group achieved statistical significance. Our hypothesis has also been endorsed by findings of a MedGem^®^ validation study among patients with anorexia nervosa, which reported individual RQ varied significantly from the fixed 0.85 used by MedGem^®^ [28]. Although the use of MedGem^®^ eliminates the need to calculate dry weight and adjust for disease severity, the fixed RQ of 0.85 may not accurately reflect the metabolic changes associated with advanced cirrhosis and sarcopenia, which is consistent with a lower RQ.

### 4.1. Strengths and Limitations

Strengths of our study include a single operator for both MedGem^®^ and Vmax measurements to ensure consistency of techniques and process, adherence to testing measures, and a specific pre-liver transplant population.

A major limitation of this study was the low sample size of 14 patients, impacting the power of our study, resulting in wide mean differences and standard deviations, and unequal distribution of gender—a reflection of the broader population with a diagnosis of cirrhosis. Our study was also limited by patient heterogeneity; patients had a range of aetiologies for their liver cirrhosis, MELD scores, and presence or absence of ascites. It is possible that either the disease heterogeneity or low sample size leading to limited power may have contributed to the lack of significant findings. Despite this, our data were still normally distributed and did produce some significant findings. Our low sample size limited the ability to conduct adequately powered subgroup analyses between the SGA malnutrition classes.

We excluded data from one participant due to highly discrepant values using Vmax. We could not identify any features of this participant, including deviation from standard testing protocol, to account for the discrepant findings. It is possible that there was an error in Vmax testing for this patient, as the measured REE by Vmax was significantly higher than other predictions and measures, and was not consistent with the clinical picture. This example highlights potential limitations of the reference standard, Vmax, which carries its own potential for error in REE measurement.

Our protocol followed best practice guidelines for the use of Vmax; however, there is no uniform procedure and analysis protocol available to ensure the accuracy and precision of metabolic carts [22]. This presents a limitation for using Vmax as the gold standard method to assess REE [15,21,22]. Cooper et al. identified Vmax could not be considered “adequately reliable in a research setting” [19]. This challenge, while inherent with all currently commercially available metabolic carts, may limit the quality of our data. Our protocol would have been strengthened by repeat MedGem^®^ measurements in addition to measurements of the same patients on multiple days to establish intra-device and intra-person reliability of our results.

### 4.2. Future Direction

We hope that our data will serve as a pilot for future testing of the MedGem^®^ device among patients with cirrhosis. Further testing with larger sample sizes should be carried out specifically focusing on moderately and severely malnourished patients, in patients with and without ascites, and within a more homogenous aetiology of cirrhosis. This will help identify certain patient subsets where MedGem^®^ indirect calorimetry may have better performance characteristics compared to predictive equations. Future studies should also specifically assess RQ in malnourished cirrhotic patients to further delineate if low RQ affects the reliability of the MedGem^®^ REE.

## 5. Conclusions

To avoid further exacerbating malnutrition or overfeeding adequately nourished cirrhosis patients, reliable, valid, and feasible methods to calculate REE and overall nutrition prescription are needed. Current available methods have poor levels of agreement and should not be considered a definitive guide for prescribing nutrition interventions in these patients. Patients should be followed closely to identify if their nutrition prescription is meeting or exceeding their needs over time.

## Figures and Tables

**Table 1:** Demographic data of 14 participants.

Variable	Result
SGA Score	
A, mild (%)	5 (36)
B, moderate (%)	5 (36)
C, severe (%)	4 (29)
Gender	
Male (%)	10 (71)
Female (%)	4 (29)
Age (years)	53.5 ± 9.4
Height (cm)	167.7 ± 8.8
Estimated dry weight (kg)	65.1 ± 8.1
Estimated dry BMI (kg/cm^2^)	
Males	22.6 ± 2.8
Females	24.5 ± 2.4
Aetiology of cirrhosis *	
Hepatitis C (%)	5 (36)
Alcohol (%)	3 (21)
Primary sclerosing cholangitis (%)	3 (21)
Hepatocellular carcinoma (%)	3 (21)
Non-alcoholic steatohepatitis (%)	2 (14)
Hepatitis (%)	1 (7)
Budd Chiari syndrome (%)	1 (7)
MELD score	12.3 ± 4.2
Ascites/severity (*n*)	
None (%)	9 (64)
Mild (%)	1 (7)
Moderate (%)	1 (7)
Severe (%)	3 (21)

Data are presented as mean ± standard deviation (SD) or number (%). * 4/14 have two aetiologies of cirrhosis. Abbreviations: SGA = subjective global assessment of malnutrition status; BMI = body mass index; MELD = model for end stage liver disease.

**Table 2:** Measured REE using four methods in 14 participants.

Method	REE (kcal/day)
Vmax	1472.3 ± 279.3
MedGem^®^	1452.9 ± 386.0
HBE (calculated)	1412.8 ± 165.4
MSJ (calculated)	1381.7 ± 168.3

Values are mean ± SD. MedGem–Vmax ρ_C_ = 0.80, 95% CI 0.55–0.92; HBE–Vmax ρ_C_ = 0.56, 95% CI 0.19–0.79; MSJ–Vmax ρ_C_ = 0.47, 95% confidence interval (CI) 0.07–0.75. Abbreviations:REE = resting energy expenditure; HBE = Harris Benedict equation; MSJ = Mifflin St. Jeor equation; ρ_C_ = Lin’s concordance coefficient.

**Table 3:** Mean REE using different devices compared to Vmax.

Comparison Group	Mean kcal Difference (95% CI)	Lin’s Concordance Correlation Coefficient (ρC) (95% CI)	Mean Underpredicted kcal Differences (% difference)	Mean Overpredicted kcal Differences (% difference)
MedGem^®^–Vmax	−21.4 (−143.3–100.4)	0.80 (0.55–0.92)	−188.4 ± 110.8 (*n* = 7) (13%)	145.6 ± 138.2 (*n* = 7) (10%)
HBE–Vmax	−61.5 (−182.3–59.2)	0.56 (0.19–0.79)	−220.3 ± 112.2 (*n* = 8) (18%)	150.2 ± 44.9 (*n* = 6) (9%)
MSJ–Vmax	−92.6 (−223.6–38.4)	0.47 (0.07–0.75)	−261.1 ± 132.3 (*n* = 5) (20%)	132.1 ± 58.1 (*n* = 9) (8%)

Values presented as mean ± SD. Abbreviations: REE = resting energy expenditure; CI = confidence interval; HBE = Harris Benedict equation; MSJ = Mifflin St. Jeor equation.
